# Genetic relationship between purebred and synthetic pigs for growth performance using single step method

**DOI:** 10.5713/ajas.20.0261

**Published:** 2020-08-30

**Authors:** Joon Ki Hong, Kyu Ho Cho, Young Sin Kim, Hak Jae Chung, Sun Young Baek, Eun Seok Cho, Soo Jin Sa

**Affiliations:** 1 National Institute of Animal Science, Rural Development Administration, Cheonan 31000, Korea

**Keywords:** Synthetic Pigs, Genetic Correlation, WooriHeukDon, Residual Feed Intake, Growth

## Abstract

**Objective:**

The objective of this study was to estimate the genetic correlation (*r*_pc_) of growth performance between purebred (Duroc and Korean native) and synthetic (WooriHeukDon) pigs using a single-step method.

**Methods:**

Phenotypes of 15,902 pigs with genotyped data from 1,792 pigs from a nucleus farm were used for this study. We estimated the *r*_pc_ of several performance traits between WooriHeukDon and purebred pigs: day of target weight (DAY), backfat thickness (BF), feed conversion rate (FCR), and residual feed intake (RFI). The variances and covariances of the studied traits were estimated by an animal multi-trait model that applied the Bayesian inference.

**Results:**

*r*_pc_ within traits was lower than 0.1 for DAY and BF, but high for FCR and RFI; in particular, *r*_pc_ for RFI between Duroc and WooriHeukDon pigs was nearly 1. Comparison between different traits revealed that RFI in Duroc pigs was associated with different traits in WooriHeukDon pigs. However, the most of *r*_pc_ between different traits were estimated with low or with high standard deviation.

**Conclusion:**

The results indicated that there were substantial differences in *r*_pc_ of traits in the synthetic WooriHeukDon pigs, which could be caused by these pigs having a more complex origin than other crossbred pigs. RFI was strongly correlated between Duroc and WooriHeukDon pigs, and these breeds might have similar single nucleotide polymorphism effects that control RFI. RFI is more essential for metabolism than other growth traits and these metabolic characteristics in purebred pigs, such as nutrient utilization, could significantly affect those in synthetic pigs. The findings of this study can be used to elucidate the genetic architecture of crossbred pigs and help develop new breeds with target traits.

## INTRODUCTION

The breeding objective of nucleus swine farms is to improve commercial pig performance. Breeders select purebred pigs because they assume there is a strong relationship between performance of the purebred pigs and their progenies. However, the accuracy of this selection depends on the genetic correlation (*r*_pc_) between purebred and crossbred performance. The three main components that affect this performance are genotype-by-genotype interactions, genotype-by-environment interactions, and the different definitions of traits can reduce the *r*_pc_ [1]. Synthetic pigs originated from purebred pigs; however, with increasing generations, they may develop genetically different characters, as has been observed in Iberian pigs [2]. Several studies on *r*_pc_ concentrated on the relationship with crossbred performance [3–6]. This information can be used to verify the genetic influence of purebred pigs on synthetic pigs, which helps elucidate the crossbred genetic architecture underlying complex traits. This verification can also be used to help develop new synthetic populations and improve target traits.

WooriHeukDon pigs are a synthetic breed that was pro duced by crossing Duroc with Korean native pigs in Korea [7]. The *r*_pc_ of performances traits between purebred (Duroc or Korean native) and WooriHeukDon pigs may be low because WooriHeukDon pigs are now genetically distinct [7], and the number of common sires was small [1]. However, feed efficiency traits can be more complex than growth traits. Feed efficiency is related to nutrient utilization, and energy digestibility and selection, and improved feed efficiency can improve various functions that use energy and nutrients [8,9].

We assumed that, in the same feeding environment, the genetic characteristics of energy digestibility were similar between purebred and synthetic pigs. Our Duroc, WooriHeukDon, and Korean native pigs were raised at the same farm under the same feeding system. Therefore, we estimated the *r*_pc_ of several performance traits between WooriHeukDon and purebred pigs: day of target weight (DAY), backfat thickness (BF), feed conversion rate (FCR), and residual feed intake (RFI).

## MATERIALS AND METHODS

### Dataset

We used data provided by the National Institute of Animal Science in Korea. The pedigree included 13,031 Duroc, 1,529 Korean native, and 2,267 WooriHeukDon pigs (Table 1). The population structures of these breeds were determined using CFC v1.0 [10].

WooriHeukDon pigs are a synthetic breed that was pro duced by crossing Duroc with Korean native pigs in Korea. A total of 50 pure pigs (42 Duroc and 10 Korean native) were used to produce WooriHeukDon pigs from 2008 to 2010. Initially, pure Korean native boars were crossed with Duroc sows to produce F1 animals. Then, Duroc boars were used to sire the F2 generation and produced 25% Korean native and 75% Duroc pigs, which were further crossed with F1 individuals to finally yield 37.5% Korean native and 62.5% Duroc pigs. Furthermore, WooriHeukDon (37.5% Korean native and 62.5% Duroc) pigs were selected for about seven generations from 2010 to 2018.

The experimental protocols describing the management and care of the animals were reviewed and approved according to the Guide for the Care and Use of Laboratory Animals (National Institute of Animal Science, Animal Care Committee of Korea) on 19 August 2019 (approval number: NIAS 2019-1709). All pigs were raised in an experimental farm of NIAS and had a space allowance of at least 1 m^2^ with solid concrete flooring. Pigs were fed *ad libitum* and water was constantly accessible through nipple drinkers. We tested all pigs that were raised under the same facilities. The feeding program and measurement of traits were conducted in accordance with the pig testing standards of the Korean Animal Improvement Association (http://www.aiak.or.kr/eng).

The performance tests of each trait started soon after each animal reached a live body weight of 30 kg and were continued until a target weight (TW) of 90 kg (70 kg for Korean native) was attained. On average, fewer than 160 days were required to attain this TW. Average daily gain (ADG, g/d) was calculated as TW minus start weight divided by the length of the period. BF was measured using an A-mode ultrasound device (Renco Lean-Meter series 12, Renco Corporation, Minneapolis, MN, USA). Average backfat thickness (ABF) was obtained by calculating the average BF of the shoulder (on the 4th thoracic vertebrae), mid-back (on the last thoracic vertebrae), and loin (on the last lumbar vertebrae). Approximately four to six female pigs were kept in a pen. Alternatively, one or two male pigs from the same litter were kept in a pen to calculate feed intake. Feed disappearance from each pen was recorded during performance tests to calculate average daily feed intake (ADFI). FCR was calculated as ADFI divided by ADG. DAY and BF were calculated as follows [11]:

$$\begin{array}{l} \text{DAY}=\text{age at the test}+\frac{\left(\text{TW}-\text{body weight at the test})\times (\text{age at the test}-38\right)}{\text{body weight at the test}}\\ \text{BF}=\text{ABF}+\frac{\left(\text{TW}-\text{body weight at the test})\times (\text{ABF}\right)}{\left(\text{body weight at the test}-11.34\right)}\\ \text{BF }(\text{Korean native})=\text{ABF}+\frac{\left(\text{TW}-\text{body weight at the test})\times (\text{ABF}\right)}{\left(\text{body weight at the test}-4.3\right)} \end{array}$$

RFI was obtained as the difference between the observed and predicted ADFI [12]:

$$\text{ADFI}=\mu +b_{1}\text{ADG}+b_{2}\text{BF}+b_{3}{\text{AGE}}_{\text{onset}}+\text{e},$$

where AGE_onset_ is the age at which the animal was tested; *b*_1_, *b*_2_, and *b*_3_ are the linear coefficients of the regression for covariates; and *e* is the RFI. The phenotypic dataset, which included DAY, BF, FCR, and RFI, was obtained from performance tests (Table 2). Korean native pigs had records only for DAY and BF.

### Genotyping of animals

The genomic DNA of pigs was extracted from their blood samples from 2010 to 2018 using a standard protocol. A total of 1,792 pigs (881 Duroc, 204 Korean native, and 707 WooriHeukDon) were genotyped using the Illumina PorcineSNP60 v2 BeadChip panel, which comprised 61,565 single nucleotide polymorphism (SNP) markers [13]. The quality control of the SNP markers included deletion of individuals with parent–progeny Mendelian conflicts or with a missing rate of >0.90, and removal of monomorphic SNP genotypes, and SNPs located on sex chromosomes, with minor allele frequency (MAF)<0.05, genotype call rates of <0.90, and Hardy–Weinberg equilibrium of 0.15 [14]. After quality control, the final dataset contained genotypes from 1,771 pigs (878 Duroc, 196 Korean native, and 697 WooriHeukDon pigs). The total number of autosomal SNPs was reduced to 42,486.

### Genetic parameter estimation

Genetic correlation (*r*_pc_) of performance between purebred and WooriHeukDon pigs was estimated for corresponding traits (e.g., DAY in Duroc and WooriHeukDon pigs) and different traits (e.g., DAY in Duroc and RFI in WooriHeukDon pigs). Genetic (*r*_g_) and phenotypic (*r*_p_) correlations between different traits were estimated within breeds.

The variances and covariances of the studied traits were estimated by an animal multi-trait (10 traits) model that applied the Bayesian inference using GIBBS2F90 [15]. The Gibbs samplers were run as single chains of 220,000 rounds. The first 20,000 rounds were discarded as burn-in, thinning every 20 samples. This resulted in 10,000 samples being used for post-Gibbs analyses, which were completed using POSTGIBBSF90 [15].

The effects of sex (male or female) on both DAY and BF and birth year- month (134 levels) were fitted as fixed effects. The models also included the random effects of birth litter (2,790 levels). Animals were fitted as a random effect in the model. The statistical model for each group of traits was as follows:

y=Xb+Za+Wc+e,

in which ***y*** is the vector of observations; ***X***, ***Z***, and ***W*** are the corresponding incidence matrices; ***b*** is the vector of fixed effects; ***a*** is the vector of random additive genetic effects (breeding values), ***a*** ~ *N*(0, ***H*** ⊗ ∑*_a_*); ***c*** is the vector of random birth litter, ***c*** ~ *N*(0, ***I*** ⊗ ∑*_c_*); and **e** is the vector of residuals, ***e*** ~ *N*(0, ***I*** ⊗ ∑*_e_*). The **H** matrix in the single-step method defines the relationship of the genomic and pedigree information. The inverse **H** matrix is rather simple in structure [16,17] and can be written as:

$$H^{-1}=A^{-1}+\left[\begin{array}{cc} 0 & 0\\ 0 & G^{-1}-A_{22}^{-1} \end{array}\right],$$

Where the **A**_22_ matrix is only for genotyped animals, **A** is the relationship matrix among individuals based on pedigree information, and **G** is the relationship matrix among individuals based on genomic information. The **G** matrix was built according to PM VanRaden [18]. Both **A**_22_ and **G** were subsequently combined, because the two matrices were similar. **I** is an identity matrix of appropriate dimensions. Phenotype variance is the sum of random and residual variances. Heritability (*h*^2^) was calculated as the additive genetic variance divided by the phenotype variance.

## RESULTS

### Heritability estimation

Heritability (*h*^2^) obtained from the studied model is presented in Table 2. In Duroc pigs, heritability was high (*h*^2^>0.5) for RFI, and moderate (*h*^2^ = 0.2 to 0.5) for DAY, BF, and FCR. In WooriHeukDon pigs, all traits were moderately heritable. Heritability estimates in Korean native pigs were high for DAY and BF. Across the breeds, heritability estimates for DAY (0.36±0.05) and BF (0.38±0.06) in WooriHeukDon pigs were higher than those in Duroc pigs (DAY 0.31±0.03 and BF 0.30±0.03), whereas heritability estimates for FCR (0.48 ±0.08) and RFI (0.48±0.07) in WooriHeukDon pigs were somewhat lower than those in Duroc pigs (FCR 0.49 ±0.04 and RFI 0.51±0.04). Korean native pigs had nearly twice the estimated heritability compared with the other breeds for both DAY (0.57±0.06) and BF (0.76±0.06).

### Genetic correlation between traits within breeds

The *r*_g_ estimates within breeds are given in Table 3. The *r*_g_ estimates between traits were typically low to moderate for all breeds, although this was not the case for *r*_g_ between FCR and RFI. Additionally, the association of RFI with other traits was weaker than with FCR. The *r*_g_ between DAY and BF was low, and ranged from 0.07 to 0.10. In both Duroc and WooriHeukDon pigs, the *r*_g_ of DAY was moderately positive (0.25 to 0.38) with FCR but negative (−0.16 to −0.26) with RFI. BF was weakly associated (0.02 to 0.07) with FCR and RFI in Duroc pigs. Alternatively, in WooriHeukDon pigs, BF also had low *r*_g_ (0.06) with RFI but moderate *r*_g_ (0.34) with FCR. FCR was strongly positively associated (0.61 to 0.81) with RFI in both Duroc and WooriHeukDon pigs.

### Purebred–synthetic genetic correlations

The *r*_pc_ estimates are presented in in Table 4. The *r*_pc_ estimates within traits were low (less than 0.10) for DAY and BF. In Duroc and WooriHeukDon pigs, there were strong correlations for RFI and FCR; the *r*_pc_ estimates were 0.99 for RFI and 0.64 for FCR. For *r*_pc_ estimates between different traits, DAY in WooriHeukDon pigs was more correlated with both FCR and RFI than DAY in Duroc pigs. The *r*_pc_ estimates for DAY in WooriHeukDon pigs were −0.21 for FCR and −0.26 for RFI of Duroc pigs.

## DISCUSSION

### Heritability and genetic correlation within breeds

The heritability of growth and BF have been reported in many studies. In general, growth traits have moderate heritability, whereas BF has moderate to high heritability [11,19–22]. In this study, DAY and BF also had moderate heritability in Duroc and WooriHeukDon pigs (Table 2). The higher heritability estimates for DAY and BF in Korean native pigs was consistent with previously published estimates [23,24]. Estimated heritabilities for feed efficiency (FCR, RFI) were close to 0.5 in both breeds and somewhat higher than those (0.30 to 0.42) of previous studies [25–28].

Our low to moderate genetic correlations between DAY and BF in all breeds (Table 3) coincided with those of previous studies [11,22,29]. In Duroc and WooriHeukDon pigs, FCR was favorably genetically correlated with DAY and BF. This result is consistent with previous reports of FCR being negatively related to growth and positively related to BF [8,30]. However, because ratio traits such as FCR are not ideal for elucidating feed efficiency [31], RFI is a commonly used to measure feed efficiency. In this study, the genetic correlation between FCR and RFI was very high in Duroc pigs, which indicated that this correlation was greater in the sire than other lines [30]. RFI is more independent of production than FCR in Duroc and WooriHeukDon pigs. Additionally, the genetic correlations of RFI were low with BF but somewhat moderate with DAY in Duroc and WooriHeukDon pigs. Generally, RFI has a neutral relationship with other traits [8]. In particular, superior breeds for growth and lean meat, such as Pietrain pigs, had a more neutral relationship between RFI and other growth traits [30,32]. However, RFI can also have a slightly unfavorable relationship with growth [12,33–35]. RFI in WooriHeukDon pigs has a more unfavorable relationship with growth compared with that in Duroc pigs; this means that the faster WooriHeukDon pigs grow, the more they consume to maintain energy. WooriHeukDon pigs have slower growth and more backfat than Duroc pigs (Table 2) because were developed by the genetic characteristics of both Duroc and Korean native pigs. Therefore, there might be differences in the genetic relationship between RFI and growth based on breed characteristics.

### Genetic correlation between purebred and synthetic performance

The *r*_pc_ for DAY and BF were lower than 0.1 (Table 4), and these values were substantially different from those produced by other studies that estimated *r*_pc_ between purebred and crossbred performance for growth traits. Wientjes and Calus [1] reviewed existing literature that report *r*_pc_ estimates in pigs, and noted that the average *r*_pc_ was 0.66 for growth traits and 0.69 for backfat. A low *r*_pc_ may occur because of biological differences, genotype-by-environment interactions, and the different definition of the considered trait [1,35,36]. In this study, there should be no genotype-by-environment interactions for *r*_pc_ because all pigs were tested in the same environmental facility. The weighting criterion of Korean native pigs (70 kg) differed from that of WooriHeukDon pigs (90 kg), which might be one factor that contributed to the very low *r*_pc_ between the two breeds. Additionally, unlike most crossbred pigs, WooriHeukDon pigs are a product of a more complex combination of crosses and have their own generations, which could have resulted in biological differences from purebreds. Duroc and WooriHeukDon pigs are selected based on the same traits, DAY and BF. However, crossbreed genomic information is made up of a mosaic of genomic regions inherited from different breeds, and there might be different allele effects for the same trait [37]. Unlike WooriHeukDon pigs, Korean native pigs have only been selected for their genetic diversity, which could also result in different selection signals between Korean native and WooriHeukDon pigs [7].

However, the *r*_pc_ for RFI between Duroc and WooriHeukDon pigs was very strong and close to 1 (Table 4). This indicated that there could be substantial differences in *r*_pc_ of traits in synthetic pigs, unlike in other crossbred pigs. Feed efficiency is complex and includes two or more traits, and it is more essential for metabolism than other growth traits. High of *r*_pc_ values for feed efficiency have also been obtained in previous studies [5,6,35,38,39]. RFI is related to nutrient utilization and energy digestibility, and selection based on RFI could improve various functions that use energy and nutrients [8,9]. There are various reports about the potential importance of low RFI in pigs, such as high anti-oxidant defenses in the mitochondria of body [40], superior ability to cope with stress-related behavioral reactivity [41], and low levels of viremia when infected with porcine reproductive and respiratory syndrome virus [42]. These biologically important characteristics in Duroc pigs, such as nutrient utilization and disease resistance, might also be found in WooriHeukDon pigs. Because the number of similar SNP regions was related to *r*_pc_ of the trait [37], Duroc and WooriHeukDon pigs might have many common regions for RFI. The impact of breed-specific SNP effects in purebred and synthetic pigs needs further study, because our study did not estimate SNP effects for each breed.

To the best of our knowledge, this is the first report of *r*_pc_ between different traits in pigs. However, there was some limitations of our study with small data sets in both local breeds (Korean native and WooriHeukDon). Especially, the most of *r*_pc_ were estimated with high standard deviation. Therefore, our results also indicated the value of further analysis with a greater population to obtain a more robust estimation of *r*_pc_ between purebred and synthetic pigs.

## CONCLUSION

The objective of this study was to estimate *r*_pc_ of growth performance (DAY, BF, FCR, and RFI) between synthetic (WooriHeukDon) and purebred (Duroc and Korean native) pigs. The *r*_pc_ was lower than 0.1 for DAY and BF but high for FCR and RFI. In particular, *r*_pc_ of RFI was close to 1. The results indicated that there were substantial differences in *r*_pc_ of traits in the synthetic WooriHeukDon pigs, which could be caused by these pigs having a more complex origin than other crossbred pigs. We conclude that the RFI of Duroc pigs was strongly correlated with that of WooriHeukDon pigs, and these breeds might have a lot of common regions related to RFI. RFI is more essential for metabolism than other growth traits and these metabolic characteristics in purebred pigs, such as nutrient utilization, could significantly affect those in synthetic pigs. The findings of this study can be used to elucidate crossbred genetic architecture and help develop new breeds with particular target traits.

## Figures and Tables

**Table 1:** Number of animals and pedigree information of each breed

Item	Duroc	Korean native	WooriHeukDon
Birth year	1995 to 2018	1995 to 2018	2008 to 2018
Number of founders	297	29	61
Number of individuals	13,031	1,529	2,267
Longest ancestral path	19	14	10
Family (Full-sib groups) size	2 to 21	2 to 14	2 to 19
Average inbreeding coefficients (min.–max.)	0.032 (0.0004–0.315)	0.128 (0.016–0.390)	0.070 (0.023–0.348)

**Table 2:** Number of observations (N), mean, standard deviation (SD) for phenotype and heritability

Breed	Trait	N	Phenotype		Heritability		
	
			Mean	SD	Mean	SD	95% HPD1)
Duroc	DAY	12,412	138.73	11.92	0.31	0.03	0.26–0.36
	BF	12,292	12.48	1.92	0.30	0.03	0.24–0.36
	FCR	4,578	2.30	0.30	0.49	0.04	0.42–0.58
	RFI	2,307	165.47	152.05	0.51	0.04	0.43–0.59
WooriHeukDon	DAY	2,063	148.84	12.68	0.36	0.05	0.25–0.46
	BF	2,048	16.60	3.71	0.38	0.06	0.27–0.50
	FCR	551	2.36	0.39	0.48	0.08	0.32–0.64
	RFI	290	98.41	88.49	0.48	0.07	0.35–0.62
Korean native	DAY	1,427	192.42	24.70	0.57	0.06	0.45–0.69
	BF	1,414	20.58	4.09	0.76	0.06	0.65–0.87

DAY, day of target weight; BF, backfat thickness; FCR, feed conversion rate; RFI, residual feed intake.

1) 95% HPD, highest posterior density interval containing 95% of the observations.

**Table 3:** Posterior mean for genetic correlations (upper diagonal) and phenotypic correlations (lower diagonal) between traits

Breed	Trait	DAY	BF	FCR	RFI
Duroc	DAY	-	0.08 0.07	0.25 0.07	−0.16 0.08
	BF	0.120.01	-	0.07 0.07	0.02 0.07
	FCR	0.430.02	0.00 0.00	-	0.87 0.02
	RFI	−0.010.02	−0.01 0.02	0.84 0.01	-
WooriHeukDon	DAY	-	0.27 0.12	0.38 0.13	−0.26 0.18
	BF	0.200.03	-	0.34 0.14	0.06 0.17
	FCR	0.460.04	0.25 0.05	-	0.61 0.10
	RFI	−0.110.06	−0.06 0.06	0.34 0.06	-
Korean native	DAY	-	0.07 0.10	-	-
	BF	0.100.06	-	-	-

DAY, day of target weight; BF, backfat thickness; FCR, feed conversion rate; RFI, residual feed intake.

Posterior standard deviations are given as subscripts.

**Table 4:** Posterior mean for genetic correlations (*r*_pc_) between purebred and WooriHeukDon traits

Breed	Trait	WooriHeukDon			

		DAY	BF	FCR	RFI
Duroc	DAY	**0.05_0.19_**	−0.08_0.17_	−0.11_0.14_	−0.16_0.08_
	BF	−0.10_0.14_	**0.02_0.14_**	−0.02_0.11_	0.02_0.07_
	FCR	−0.21_0.20_	0.03_0.17_	**0.53_0.12_**	0.86_0.02_
	RFI	−0.26_0.18_	0.06_0.17_	0.61_0.10_	**0.99_0.00_**
Korean native	DAY	**0.07_0.12_**	0.01_0.12_	0.04_0.10_	−0.01_0.08_
	BF	0.03_0.08_	**0.01_0.08_**	0.02_0.07_	0.00_0.05_

DAY, day of target weight; BF, backfat thickness; FCR, feed conversion rate; RFI, residual feed intake.

Posterior standard deviations are given as subscripts. The *r*_pc_ within traits are given as bold.
